# Risk Factors Linked to Psychological Distress, Productivity Losses, and Sick Leave in Low-Back-Pain Employees: A Three-Year Longitudinal Cohort Study

**DOI:** 10.1155/2016/3797493

**Published:** 2016-08-18

**Authors:** Angelo Compare, Paolo Marchettini, Cristina Zarbo

**Affiliations:** ^1^Department of Human and Social Sciences, University of Bergamo, Bergamo, Italy; ^2^Human Factors and Technologies in Healthcare Research Centre, University of Bergamo, Bergamo, Italy; ^3^Pain Medicine Centre, Centro Diagnostico Italiano, Milan, Italy; ^4^Pain Medicine Centre, Ospedale San Raffaele, Milan, Italy; ^5^University of Applied Science of Southern Switzerland, Pain Pathophysiology and Therapy Programme, Manno, Switzerland

## Abstract

*Background*. Low back pain (LBP) is one of the most common health problems worldwide.* Purpose*. To investigate the link between baseline demographic and occupational, medical, and lifestyle data with following psychological and occupational outcomes in a large sample of employees with LBP over a 3-year period.* Study Design*. Three-year prospective cohort study.* Methods*. Italian-speaking employees (*N* = 4492) with a diagnosis of LBP were included. Screening at Time 1 was done in order to collect information about severity and classification of LBP, demographic, lifestyle, and occupational status data. Psychological distress (PGWBI) and occupational burden were assessed after 3 years.* Results*. After 3 years, employees with LBP not due to organic causes had an increased risk of psychological distress. Gender appears to be an important variable for following occupational burden. Indeed, being a white-collar man with a LBP without organic causes seems to be a protective factor for following work outcomes, while being a white-collar woman with a LBP not due to organic causes appears to be a risk factor for subsequent sick leave. Moreover, LBP severity affects psychological and occupational outcomes.* Conclusion*. Our findings have several implications that could be considered in preventive and supportive programs for LBP employees.

## 1. Introduction

Low back pain (LBP) is one of the most common health problems worldwide. The World Health Organization [1] states that it affects approximately 80–85% of people over their lifetime. Low back pain (LBP) has been defined as any neuromusculoskeletal disorder affecting the low back including all back pain, lumbar disk problems (displacement, rupture) and sciatica, not caused by other diseases, injuries (e.g., cancer or motor vehicle accident), or cervical spine problems (e.g., neck pain or neck torsion problems) [2]. LBP is defined as acute if it lasts from 2 to 4 weeks, subacute up to 12 weeks, and chronic for more than 12 weeks [3]. Analysing the signs during a medical examination, it is possible to classify LBP due to organic causes or without organic causes [4]. According to the evidence, patients without demonstrable organic causes of their disease tend to describe their pain as more inconstant and diffuse while those whose disease has demonstrable organic causes describe the pain as more consistent and specific [5].

In high-income countries, low back pain is the most frequent occupational health problem: approximately 2–5% of workers have chronic low back pain [6]. It is also the most frequent activity-limiting complaint in the young and middle aged population and the second leading cause of sick leave [7]. Former studies have reported sex differences in various aspects of LBP, suggesting a higher prevalence of the disease in women. Moreover, females with LBP seem to be more likely to seek care and to take sick leave than males [8–11].

The impact of LBP is multifactorial and affects several areas of subjective life, like participation restrictions, career burden, use of healthcare resources and financial burden. Moreover, several sociological, psychological, and cognitive factors have been related to high levels of LBP: job dissatisfaction, low income, leisureliness, anxiety, depression, fear-avoidance beliefs, passive coping, and self-reported feelings of disability [12–21]. In particular, it has been suggested that LBP has a clear impact on productivity at work and that its related costs can be remarkable [22–24]. Moreover, evidence shows that anxiety, depression, fear-avoidance beliefs relating to work and low back pain are predictors of impairment in subsequent physical health-related quality of life and number of healthcare contacts [25]. Furthermore, lower levels of quality of life in LBP patients were associated with efficiency loss and absenteeism [26]. For all these reasons, the financial burden of LBP is substantial and includes the costs of medical care, indemnity payment, productivity loss, employee retraining, administrative expenses, and litigation [27–29]. Even if literature describes the impact of LBP on several aspects of human life, little is known about protective and risk factors for psychological and occupational burden in this population. Previous findings on LBP workers have reported that risk factors of sick leave are being female [30], being blue collar worker [31] or a white collar [32], high physical load, and the severity of the illness [33]. On the contrary, physical exercise has shown a protective effect on musculoskeletal symptoms (MSS) [34]. Moreover, to the best of our knowledge, no studies have focused on factors associated with the following psychological distress and work performance in LBP population.

In order to overcome literature's gap, the aim of this study was to investigate the link between baseline demographic, occupational, medical, and lifestyle data with following psychological and occupational outcomes in a large sample of employees with LBP over a 3-year follow-up period. Therefore, we tested the hypothesis that baseline variables in LBP employees could predict/being risk or protective factors for following psychological distress, work performance, and sick leave.

## 2. Method

### 2.1. Procedure and Sample Selection

**Figure 1:**
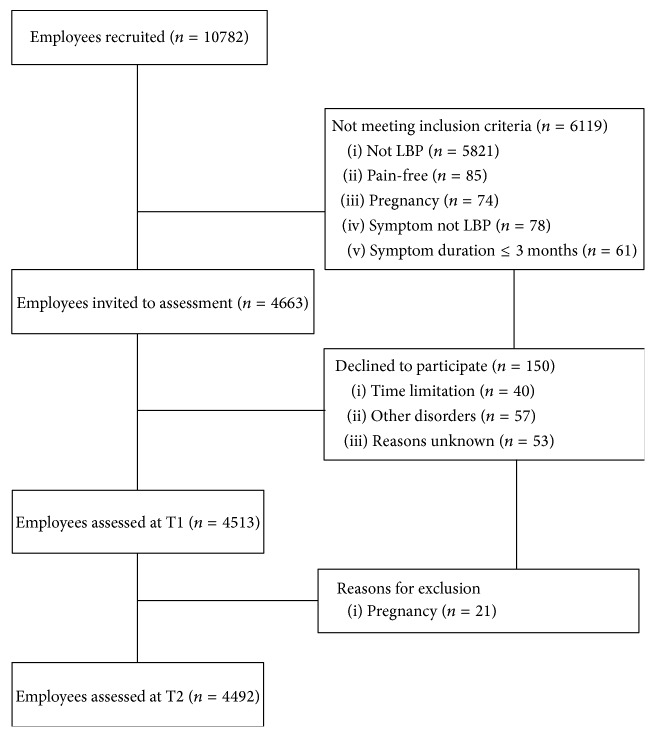
Participants flow. Note: LBP, Low Back Pain.

Participants at Time 1 (T1) were selected through the routine medical check-up enterprise program for employees and performed on 45 Italian companies in the north of Italy from 2009 to 2013. A population of 10782 employees were screened by occupational physicians. For this research study we used employee's medical information of the routine medical check-up enterprise program stored within the electronic medical records. The routine medical check-up enterprise program is repeated on the same employees every 3 years (Time 2). For the aim of this research we selected those employees that (a) received a diagnosis of LBP at both Times 1 and 2; (b) during the medical examination at Time 2 had responded affirmatively to the question “low back pain remained constant during the three years?”; and (c) fulfilling the following criteria: LBP, pain between costal margins and gluteal folds with or without referred leg pain; >12-week duration of symptoms; not pregnant; absence of spinal disorders or no other severe disorders; age between 18 and 60 years; Italian understanding and speaking. LBP diagnoses were coded by occupational physicians as “M545” (ICD-10) within the medical records. Employees selected (*N* = 4663) were contacted by e-mail and invited to enrol in the study. In order to support participation to the study a free specialist assessment of the psychological condition was proposed to each participant. 150 employees declined to participate in the study and 21 were pregnant during the assessment. Finally, a total of 4492 LBP employees accounted for the study sample (see Figure 1). The 3-year follow-up was chosen following previous similar researches [33, 35–41] and considering economic effects in the medium term. Demographic and pain data were collected using information stored in electronic medical records at T1. The mean follow-up time was 2.7 years. Collected data at T1 have been resumed in Table 1. The Regional Ethical Review Board in Milan approved the study. Written informed consent was obtained from all individuals.

### 2.2. Measures

Participants were assessed by an occupational physician at both times for sociodemographic data, back pain history, comorbidity, and pharmacological treatment. Moreover, pain intensity, previous LBP (y/n), drug treatment for LBP (y/n), and physical activity (y/n) were assessed. Drug treatment for LBP was evaluated verifying if patient had been treated by medications for LBP, in accordance with the international guidelines [42, 43], looking at the electronic health records and by checking with the patient-reported. Physical activity was assessed following the “recommended levels of physical activity for adults aged 18–64 years” of Word Health Organization [44] by patient-reported. Physical activity was assessed as yes if these criteria were verified: 150 minutes of aerobic physical activity throughout the week; aerobic activity performed for a duration of, at least, 10 minutes; muscle-strengthening activities on 2 or more days a week. At T2 psychological distress and work performance were assessed by a web platform. Also the number of sickness absences has been collected as outcome measures at follow-up.

#### 2.2.1. Sociodemographic Measures

The occupational physician collected additional data during the medical check-up: civil status (single/separated/divorced/widowed; married/common/law partner); work role (blue collar/white collar worker). Further information was gathered from the payroll database of the companies: annual personal income (≤10,000; 10,001–20,000; 20,001–30,000; 30,001–40,000; 40,001–50,000; 50,001–70,000; ≥70,001); industrial sector (banking/finance; insurance; manufacturing; ICT; public services); last level of education completed (primary; high school; university).

#### 2.2.2. Pain Severity and Organic versus Not-Organic Causes of LBP

Pain severity was measured using the Back Pain Intensity (BPI) subscale. The BPI has been shown to be a reliable and valid measure of pain for use in LBP populations [45]. The BPI subscale asks patients to rate their current pain intensity and also their pain over the last week, using a numeric scale of 0–10. Scales are anchored with the phrases ‘‘no pain” and ‘‘pain as bad as you can imagine”; these ratings are combined to give a composite index of pain severity [46].

Waddell et al.'s guidelines [4] were used to distinguish between organic (OC) and not-organic (n-OC) causes for LBP. Waddell guidelines are organized in five categories of physical signs, indicative of not-organic or psychological component to low back pain. The five categories include tenderness signs (superficial/not-anatomical); stimulation signs (axial loading/rotation); regional signs (weakness/sensory changes); distraction signs (straight leg raising); overreaction. LBP with Orthopaedic Objective Examination in the norm was established as criterion to identify not-organic (n-OC) causes of LBP.

#### 2.2.3. Target Outcomes Variables

*Psychological Distress*. Psychological distress was evaluated using the Italian version of the Psychological General Well-Being Index (PGWBI) [47, 48] at T2. PGWBI is a reliable and valid self-reporting questionnaire that allows measuring subjective psychological distress over the previous 4 weeks [49]. It includes 22 items that provide a total score and subscores for each of the following six dimensions: anxiety, depressed mood, positive well-being, self-control, general health, and vitality. The subject rates each item on a six-point Likert scale (5 is the most positive option and 0 is the most negative). The total score can range from 0 to 110, with higher scores representing the best achievable level of well-being [47]. PGWBI has been widely used in different psychosomatic studies and has shown good correlation with medical markers of chronic stress condition [50–52]. The PGWBI has been correlated with a large number of other indices of physical and mental health [53–56]. The internal consistency showed high values of the Cronbach's alpha coefficient (range 0.80–0.92) [48]. In this study, Cronbach's alphas of PGWBI global score and anxiety, depressed mood, positive well-being, self-control, general health, and vitality subscales were, respectively, 0.93, 0.90, 0.89, 0.91, 0.87, 0.92, and 0.92.

*Sick Leave*. The number of sickness absences occurring during the period from T1 and T2 was used as outcome. This outcome effectively uses the information when one individual had several sickness absence and is not dominated by only a few prolonged nonattendances at work. Over the follow-up period, information on the frequency and duration of spells of sick leave were gathered for each respondent and all consecutive sick leave spells were combined. Diagnoses for the sick leave spells were unavailable. Absence for other reasons than sickness was subtracted (e.g., absence to take care of a sick child). In this study self-certified sickness leave included absences lasting 1−3 days and medically certified 4 days or longer. Participants could have both types of sick leave spells.

*Work Performance*. According to international guidelines [57], work performance, called also presenteeism, can be conceptualized as the productivity loss without work absences. Productivity loss was assessed at T2 using the “Quantity and Quality” method [57] by the module D of the Productivity and Disease Questionnaire (Prodisq) [58]. The Prodisq has been developed based on the Quantity and Quality (QQ) method and provides a reliable and valid tool for measuring quantity and quality of work on a daily basis. Subjects were asked on average the degree (%) of reduced daily work performance due to LBP. Healthy baseline performance was assumed to be 100% productivity. The respondents gave their marks for the quality and the quantity of their work on the last working 3 years on a visual analogue scale. Subsequently the productivity loss in hours during paid work has been calculated as follows:

(1) Work  performance=1−quality10×quantity10×working  hours  per  day×days  worked.

In this study the relative productivity loss that is the productivity loss divided by the number of working hours per day, ranging from 0 (no productivity loss) to 1 (complete productivity loss), was used.

### 2.3. Statistical Analysis

Multivariate regression analyses were performed to analyse predictors of work status at 3-year follow-up. Firstly, a univariate regression was performed to assess the association between each of the independent variables and the target outcomes variables. Secondly, a forward stepwise regression including the independent variables with significant association to target outcomes variables was performed. We chose the stepwise regression selection method because it allows obscuring the independent effect of the regressors. The area under the receiver operating characteristic curve (AUC) was performed in order to validate the multiple regression model. The Wald statistics and odds ratio with 95% confidence interval (CI) were calculated for each of the predictors. The level of significance was *p* < 0.05. The SPSS Windows version 15.0 and 18.0 were used for all statistical analyses.

## 3. Results

**Figure 2:**
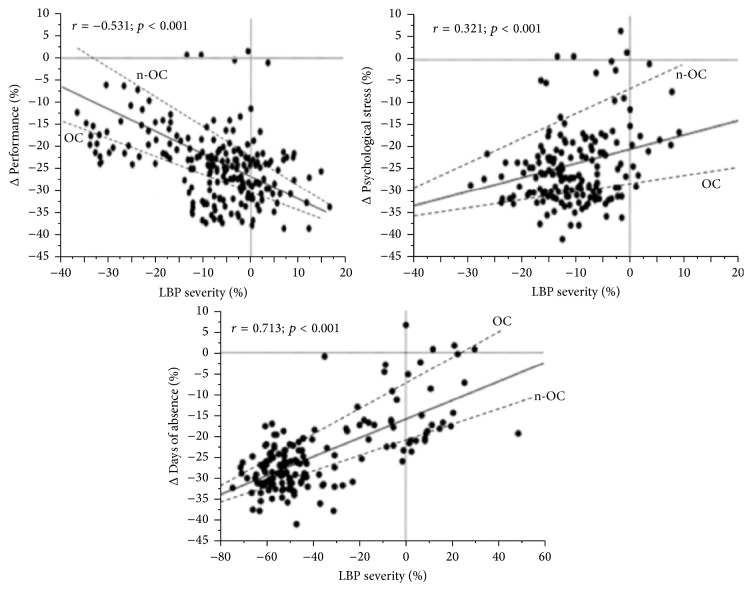
Regression plot of changes between baseline and 3-year follow-up changes in LBP severity and performance, or psychological distress, or days of absence for sick leave in the two groups (organic versus not-organic LBP). Note: LBP, Low Back Pain; OC, organic causes; n-OC, not-organic causes.

The mean relative efficiency loss due to low back pain was 0.22 (standard deviation: 0.21). The number of sickness absences from LBP represented 49% of the total days of sickness absence during 3 years. Results showed that employees with LBP due to not-organic causes are at increased risk of psychological distress (*r* = 0.321; *p* < 0.001) and greater reduction in work performance (*r* = −0.531; *p* < 0.001), while those with LBP due to organic causes showed an increase in absence from work due to illness (*r* = 0.713; *p* < 0.001) after 3-year follow-up (Figure 2). A repeated analysis controlling for gender confirmed these findings.

The univariate regression analysis for each of the predictors (Table 2) showed that gender, work roles, and lifestyle variables were significantly associated with both sick leave and work performance at follow-up. In particular, white collar (odd ratio: 0.3, CI, 0.1–0.9; *p* < 0.001), male gender (odd ratio: 0.3, CI, 0.2–0.4; *p* < 0.001), and not-organic causes of LBP (odd ratio: 0.3, CI, 0.2–0.5; *p* < 0.01) were lower risk factors for work performance, while physical activity was higher risk factor for work performance (odd ratio: 1.4, CI, 0.5–1.9; *p* < 0.01). For sick leave, physical activity was lower risk factor (odd ratio: 0.3, CI, 0.0–0.6; *p* < 0.001) while white collar role (odd ratio: 2.1, CI, 2.6–3.8; *p* < 0.01), female gender (odd ratio: 1.8, CI, 2.1–3.1; *p* < 0.0001), and not-organic causes of LBP (odd ratio: 1.8, CI, 2.6–3.6; *p* < 0.01) were higher. Blue collar role, civil status, drug treatment, LBP with organic causes, education, income, and industrial sector were not predictive factors of work performance or sick leave outcomes.

**Figure 3:**
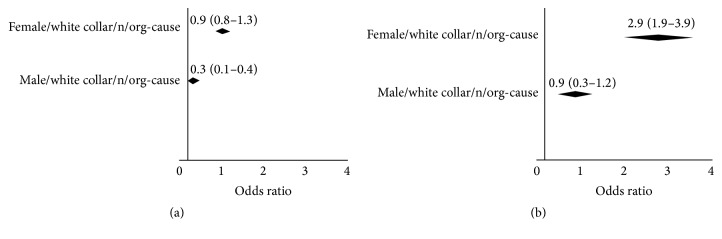
Stepwise regression and predicted values for sick leave (b) and for work performance (a) after 3-years follow-up.

Forward stepwise regression analysis was performed including the independent variables with significant association with work performance and sick leave. In the forward stepwise regression model (Figure 3), gender, white collar role, and not-organic causes of LBP were identified as predictors of the target outcomes variables. In particular, combination of male gender/white collar role/not-organic cause of LBP (odd ratio: 2.9, CI, 1.9–3.9) were higher risk profile for sick leave, while combination of male gender/white collar role/not-organic cause of LBP (odd ratio: 0.3, CI, 0.1–0.4) were lower risk profile for work performance. This model accounted for, respectively, 71% and 76% of the variance (Nagelkerke *R* ^2^) of the dependent variable. The AUC was 0.93 (95% CI, 0.81–0.98; *p* < 0.001).

## 4. Discussion

In this prospective cohort study we sought to study predictive and risk/protective factors for psychological distress, work performance, and sick leave in a sample of LBP employees after 3-year follow-up. Main results will be discussed in the following sections.

### 4.1. Not-Organic Cause of LBP Severity Predicts Psychological Distress

We found that psychological distress was predicted by not-organic cause of LBP severity. Higher LBP severity seems to cause higher psychological distress, in terms of anxiety, depression, pessimism, reduced levels of self-control, and vitality, after 3-year follow-up. Interestingly, this interaction did not occur in patients with organic LBP. Our results do not confirm previous studies that stated that no differences exist between organic versus functional LBP patients in mental disturbance [59].

However, even if LBP is almost never fatal, it seems to affect individual functioning and has major implications for the quality of life. Subjects with LBP, if compared to subjects without LBP, show higher psychological distress such as anxiety, depression, and somatization [60]. Moreover, it has been suggested that patients without demonstrable disease's explanation exhibit frequent comorbid psychiatric disorders [61]. The link between not having a medical explanation of the disease and psychological distress may be due to the lack of specific treatment, unperceived support, or previous mental vulnerability. Evidence showed that psychosocial variables, generally, have more impact than biomedical or biomechanical factors on back pain disability [16]. Moreover, low levels of quality of life in LBP workers affect productivity, efficiency, and absenteeism at work [22–24, 26].

### 4.2. Protective Factors for Sick Leave and Work Performance

Our results showed that factors linked to reduction of sick leave and improvement of work performance are male gender/white collar role/not-organic cause of LBP. Physical activity seems to be linked only to the reduction of sick leave. These results indicate that male employees having not-organic cause of LBP, and who perform managerial or administrative work (white collar workers), are more likely to have an increase of productivity and lower sick leave at the 3 years of follow-up.

For cultural and personality trait reasons, white collar men could be more likely to maintain a good work performance and avoid sick leave. It is possible that our results are due to specific characteristics of this sample: high motivation to job, tenacity, high sense of responsibility, and leadership. Previous studies have shown that work, life style, health behaviours, and health condition of male white collar workers are strongly affected by the type of job and position [62]. Moreover, the lack of organic cause could lead them to not perform the so-called “sick role,” preventing then the risk of performance loss and sick leave. Indeed, a person who falls ill usually adheres to the specifically patterned social role of being sick [63].

Our results showed that engaging in physical activity has positive effects on the number of sick leaves in LBP patients. These results could be due to the well-established protective effects of healthy lifestyles (e.g., engaging in moderate physical activity) against medical diseases and psychological distress [64]. Our results confirm previous studies that have suggested that higher engaging in recreational physical activities is related to lower low back pain, disability, and psychological distress [65].

### 4.3. Risk Factors for Sick Leave and Work Performance

Sick leave seemed to be associated with female, white collar, LBP without organic causes while work performance loss seems to be associated with physical activity. Our data confirm previous studies that have shown that women with LBP are more likely to seek care and to take sick leave than men [8–11]. The sick role attributed by the female employees to themselves could lead to a focus on the illness, its negative consequences and its cure, leading in turn to negative mood (such as lack of locus of control, depression, anxiety, and fear), increased leave for sick reasons, and performance loss. Moreover, our results about the link between physical activity and work performance loss disconfirm previous results that suggested that higher levels of physical activity are related to higher quality of work performance [66].

## 5. Conclusion

We found that psychological distress is predicted by LBP due to not-organic causes of severity, suggesting that the distinction between organic and not-organic LBP is significant in the prediction of psychological outcomes. Moreover, our results showed that male, white collar, with not-organic cause of LBP employees had an improvement in work performance and a reduction of sick leave after 3 years. Conversely, being female, white collar, with not-organic cause of LBP is associated with following reduction of work performance and increased risk of sick leave. Moreover, sick leave reduction seems to be associated with physical activity.

However, the present study has several limitations that undermine the value of our findings. Majors limitations of this study were as follows: (a) the lack of control of the reasons of sick leave during the 3 years and the absence of a baseline assessment of psychological distress; indeed, sick leave probably was not only due to LBP and the possibility of comorbidity was not considered; (b) the lack of control of maintenance of the severity of LBP during the three years; (c) the lack of a measure of pain categorization useful to assess chronic pain; and (d) the 45 companies analysed not being a representative sample of the national industry sectors. Moreover, the prospective methodological design and the psychological evaluation done by web platform could represent limitations of the study.

In conclusion, despite the above cited limitations, these study's findings have several implications that could be considered in preventive and supportive programs for LBP employees. For example, physical activity in individuals with LBP could be promoted, and specific psychological support could be provided to individuals with LBP due to not-organic cause. Keeping into account risk and protective factors could result in improved psychological wellbeing, performance quality, and reduced economic burden. However, further studies methodologically stronger are required to better explain and confirm these findings.

## Figures and Tables

**Table 1:** Measurement values at the first assessment. Data are presented as mean ± SD or as number of cases (%).

	LBP (*N* = 4513)	
	*n*	%
*Blue collar, N (%)*	1978	44
*Age (years)*	46.0 ± 9.0	
*Gender, N (%)*		
Men	2576	57
Women	1937	43
*Civil status, N (%)*		
Single/separated/divorced/widowed	1440	32
Married/common/law partner	3073	68
*Last level of education completed, N (%)*		
Primary	1127	25
High school	1902	42
University	1484	33
*Annual personal income, N (%) *		
≤10,000	91	2
10,001–20,000	537	12
20,001–30,000	946	21
30,001–40,000	1130	25
40,001–50,000	632	14
50,001–70,000	679	15
≥70,001	498	11
*Industrial sector, N (%)*		
Banking/finance	1175	26
Insurance	493	11
Manufacturing	1442	32
ICT	950	21
Public services	453	10

LBP: low back pain; no-LBP: patients without low back pain diagnosis.

**Table 2:** Univariate regression analyses showing the odds ratio for work performance and sick leave at the 3-year follow-up.

	Work performance		Sick leave	
	*p*	Odds ratio (95% CI)	*p*	Odds ratio (95% CI)
*Withe collar, N (%)*	**<0.001**	**0.3 (0.1–0.9)**	**<0.01**	**2.1 (2.6–3.8)**
*Blue collar, N (%)*	0.071	1.1 (0.9–1.3)	0.062	0.9 (0.8–1.1)
*Age (years)*	0.062	0.89 (0.78–1.0)	0.076	0.93 (0.86–1.0)
*Gender, N (%)*	0.052	0.81 (0.72–1.0)	0.78	0.91 (0.83–1.1)
Men	**<0.001**	**0.3 (0.2–0.4)**	0.061	0.88 (0.80–1.0)
Women	0.071	0.96 (0.92–1.0)	**<0.0001**	**1.8 (2.1–3.1)**
*Civil status, N (%)*	0.21	0.89 (0.68–1.1)	0.069	0.94 (0.82–1.0)
*Physical activity *	**<0.01**	**1.4 (0.5–1.9)**	**<0.001**	**0.3 (0.0–0.6)**
*Organic causes of LBP*	0.061	0.8 (0.7–1.0)	0.067	0.8 (0.6–1.0)
*Not-organic causes of LBP*	**<0.01**	**0.3 (0.2–0.5)**	**0.001**	**1.8 (2.6–3.6)**
*Drug treatment for LBP*	0.053	0.8 (0.6–1.1)	0.052	0.9 (0.5–1.2)
*Previous LBP*	0.1	1.1 (0.9–0.8)	0.053	1.0 (0.5–1.3)
Single/separated/divorced/widowed	0.066	0.81 (0.71–1.0)	0.062	0.90 (0.81–1.0)
Married/common/law partner	0.067	0.76 (0.68–1.1)	0.064	0.91 (0.80–1.1)
*Last level of education completed, N (%)*	0.088	0.91 (0.7–1.0)	0.072	0.94 (0.79–1.0)
Primary	0.072	0.85 (0.79–1.1)	0.061	0.97 (0.89–1.1)
High school	0.42	0.93 (0.81–1.0)	0.060	0.98 (0.88–1.0)
University	0.061	0.86 (0.81–1.0)	0.064	0.94 (0.90–1.0)
*Annual personal income, N (%)*	0.069	0.88 (0.67–1.2)	0.065	0.95 (0.92–1.1)
≤10,000	0.063	0.82 (0.65–1.0)	0.065	0.95 (0.91–1.0)
10,001–20,000	0.058	0.99 (0.89–1.0)	0.082	0.96 (0.90–1.0)
20,001–30,000	0.056	0.91 (0.88–1.2)	0.071	0.89 (0.85–1.0)
30,001–40,000	0.052	0.88 (0.68–1.0)	0.067	0.90 (0.89–1.1)
40,001–50,000	0.062	0.99 (0.97–1.0)	0.082	0.92 (0.88–1.0)
50,001–70,000	0.061	0.89 (0.57–1.0)	0.069	0.90 (0.85–1.0)
≥70,001	0.067	0.87 (0.69–1.1)	0.54	0.91 (0.84–1.0)
*Industrial sector, N (%)*	0.069	0.81 (0.65–1.0)	0.064	0.99 (0.81–1.0)
Banking/finance	0.059	0.89 (0.78–1.0)	0.067	0.97 (0.76–1.2)
Insurance	0.071	0.76 (0.54–1.1)	0.086	0.96 (0.78–1.0)
Manufacturing	0.062	0.88 (0.49–1.0)	0.073	0.93 (0.87–1.1)
ICT	0.061	0.89 (0.84–1.0)	0.062	0.93 (0.78–1.0)
Public services	0.068	0.87 (0.82–1.1)	0.062	0.91 (0.88–1.1)

Note: LBP: low back pain; no-LBP: patients without low back pain diagnosis; *p* values < 0.05 are indicated in bold.
